# A Comparison Study of Machine Learning (Random Survival Forest) and Classic Statistic (Cox Proportional Hazards) for Predicting Progression in High-Grade Glioma after Proton and Carbon Ion Radiotherapy

**DOI:** 10.3389/fonc.2020.551420

**Published:** 2020-10-30

**Authors:** Xianxin Qiu, Jing Gao, Jing Yang, Jiyi Hu, Weixu Hu, Lin Kong, Jiade J. Lu

**Affiliations:** ^1^ Shanghai Engineering Research Center of Proton and Heavy Ion Radiation Therapy, Shanghai, China; ^2^ Department of Radiation Oncology, Shanghai Proton and Heavy Ion Center, Shanghai, China; ^3^ Department of Radiation Oncology, Shanghai Proton and Heavy Ion Center, Fudan University Cancer Center, Shanghai, China

**Keywords:** high-grade glioma, random survival forest, machine learning, particle beam radiotherapy, predictive analytics

## Abstract

**Background:**

Machine learning (ML) algorithms are increasingly explored in glioma prognostication. Random survival forest (RSF) is a common ML approach in analyzing time-to-event survival data. However, it is controversial which method between RSF and traditional cornerstone method Cox proportional hazards (CPH) is better fitted. The purpose of this study was to compare RSF and CPH in predicting tumor progression of high-grade glioma (HGG) after particle beam radiotherapy (PBRT).

**Methods:**

The study enrolled 82 consecutive HGG patients who were treated with PBRT at Shanghai Proton and Heavy Ion Center between 6/2015 and 11/2019. The entire cohort was split into the training and testing set in an 80/20 ratio. Ten variables from patient-related, tumor-related and treatment-related information were utilized for developing CPH and RSF for predicting progression-free survival (PFS). The model performance was compared in concordance index (C-index) for discrimination (accuracy), brier score (BS) for calibration (precision) and variable importance for interpretability.

**Results:**

The CPH model demonstrated a better performance in terms of integrated C-index (62.9%) and BS (0.159) compared to RSF model (C-index = 61.1%, BS = 0.174). In the context of variable importance, CPH model indicated that age (P = 0.024), WHO grade (P = 0.020), IDH gene (P = 0.019), and MGMT promoter status (P = 0.040) were significantly correlated with PFS in the univariate analysis; multivariate analysis showed that age (P = 0.041), surgical completeness (P = 0.084), IDH gene (P = 0.057), and MGMT promoter (P = 0.092) had a significant or trend toward the relation with PFS. RSF showed that merely IDH and age were of positive importance for predicting PFS. A final nomogram was developed to predict tumor progression at the individual level based on CPH model.

**Conclusions:**

In a relatively small dataset with HGG patients treated with PBRT, CPH outperformed RSF for predicting tumor progression. A comprehensive criterion with accuracy, precision, and interpretability is recommended in evaluating ML prognostication approaches for clinical deployment.

## Introduction

High-grade glioma (HGG), including WHO grade III and IV class, is the most common and lethal primary cancer in central nervous system (1). Particle beam (e.g., proton and carbon ion) radiotherapy (PBRT), with both biological and physical advantages (2, 3), can potentially improve the outcome of HGG. Our recent results showed promising efficacy of PBRT in HGG (4). However, the inherent high heterogeneity of HGG, as the dominant factor contributing to general poor treatment efficacy, induces markedly variation of individual outcome (5–7). Adequate outcome prediction, particularly at individual level, is essential but remains challenging for developing precision strategy of PBRT for HGG.

Machine learning (ML), a branch of artificial intelligence, has been employed to predict prognosis in a variety of cancer types. Noticeably, series of studies applying ML algorithms to predict the survival of HGG under standard photon-based radiotherapy have reported good performance in recent years (8–13). However, it is still controversial that which methods among ML algorithms and conventional modeling can achieve better performance in survival analysis, particularly in terms of time-to-event censored data (14–16). Hence, it is a critical need to explore which model can contribute to higher accuracy and precision of survival prediction at patient-level for HGG with PBRT.

The most typical and commonly used model of ML and conventional statistics for cancer censored survival data are random survival forest (RSF) and Cox proportional (CPH), respectively. The RSF is an ensemble ML method constructed with numerous independent decision trees, each of which receives a random subset of samples and randomly selects a subset of variables at each split in the tree for prediction. The final prediction results of a RSF model are the average of the prediction of each individual tree. The CPH model is a well-recognized statistical technique to explore the correlation between the survival time and covariates.

To our knowledge, there was no study to explore whether conventional statistics and ML method differ in the ability to predict progression or survival for HGG patients treated with PBRT. Therefore, we retrospectively collected important clinical characteristics of HGG patients underwent PBRT, as well as fundamental molecular markers and treatment information. Then, all HGG patients were randomly split into training set or testing set, and CPH model and RSF model were compared with their performance to predict progression-free survival (PFS). The model with superior performance was then utilized to build a nomogram as in individual prediction tool of progression for HGG patient underwent PBRT.

## Methods and Materials

### Study Population and Data

Institutional review board (IRB) approval was obtained from the Shanghai Proton and Heavy Ion Center (SPHIC) prior to conducting this study. Variables from three categories: patient-related, disease-related and treatment-related information was retrospectively collected. Patient-related data collected included age, gender, and Karnofsky Performance Score (KPS). Disease-related features included tumor location that was classified as invasion of subventricular zone (SVZ) or non-SVZ (17) invasion and molecular markers, including Isocitrate dehydrogenase (IDH) gene and O[6]-methylguanine-DNA methyltransferase (MGMT) promoter status. Treatment-related information consisted of surgical completeness that was divided into gross-total resection (GTR) and non-GTR (subtotal resection, partial resection, and biopsy), and the target volume for PBRT.

### Particle Radiotherapy

Conventional MR was fundamental images for radiation planning of HGG. The l-[methyl-()11C]methionine (MET)/O-(2-[18F]fluoroethyl)-L-tyrosine (FET)-positron emission tomography (PET) was optional and further required after the latest escalating boost trial initiated. In the case of incomplete resection, dose escalation trials utilizing proton followed by carbon-ion boost were encouraged to target residual lesion. Doses of PBRT were measured by Gray Relative Biological Equivalent (GyE) to account for the RBE differences compared to photon beam The clinical target volume (CTV) of high risk (CTVhr) was defined as gross-tumor volume (GTV) in residual lesion detected on imaging studies and surgical bed plus 5-mm expansion, and the CTV for lower risk (CTVlr) consisted of GTV plus 15-mm margin and edema area. The standard protocol of PBRT for all patients was CTVhr with proton beam to 60 GyE, and CTVlr with proton beam to 50 GyE.

### Statistical Analysis and Modeling Process

Progression-free survival (PFS) time is defined as the duration between the time of diagnosis and the date of progression. The Response Assessment in Neuro-Oncology (RANO) criteria (18) with interpretation modifications (19), including parameters for changes in T1-weighted enhancing lesion and non-enhancing T2/fluid attenuated inversion recovery (FLAIR) lesion, were used to determine disease progression.

The statistical analysis was performed using the R software. Baseline differences between the training set and testing set were assessed using the Mann-Whitney U test for continuous variables. Survival curves were plotted using the Kaplan-Meier method and compared using log-rank test.

Prior to constructing CPH and RSF models, the data set was split into two mutually exclusive sets. Nearly 80% of the entire dataset was assigned as the training set, which was utilized to generate the prediction model. The remaining 20% of the data was designated as the testing set, for use in estimating the model’s accuracy. During this procedure, a five-fold cross-validation that putted the dataset stratified by progression status and then sorted by survival time was performed for the purpose that the number of patients with progression and the range of survival time should be (roughly) equal across all folds. By creating folds in this way models would be tested on dataset that was mostly representative of what they saw in the training data.

CPH and RSF models were trained using the *RandomForestSRS* and *survival* R packages, respectively. The hyperparameter tuning of RSF model was performed with five-fold cross-validation on the training set. In particular, the RSF model, as an extension of random forest (RF) that ensembles tree method for analyzing time-to-event data, must select two central hyperparameters: number of randomly drawn candidate variables (*mtry*) and number of trees. Given several studies on the influence of hyperparameters on RF model regarding performance and variable importance, *mtry*= or *mtry*=*p*/3 for regression with *p* being the number of predictor variables is reasonable (20, 21). As our dataset contained 10 predictor variables, the *mtry* was set to 3. Considering the number of trees, two studies using real datasets show that 100 trees can often achieve the biggest gain of RF model performance (22, 23). Thus, the present study used 100 trees for RSF approach.

Predictive performance of model was measured with five-fold cross-validation by discrimination and calibration *via* the *pec* R package. The concordance index (C-index), which ranges from 0.5 (random prediction) to 1 (perfect prediction), reflects the discrimination power to rank individuals from low to high risk. The brier score (BS) is a metric of calibration, with lower value representing improved model accuracy. A final nomogram was developed using the method with the greatest predictive accuracy for individualized estimation of survival.

## Results

### Demographics, Clinical Characteristics, and Treatment of Patients

The entire study cohort consisted of 82 consecutive HGG patients, who underwent PBRT at Shanghai Proton and Heavy Ion Center, between 6/2015 and 11/2019. All 82 patients underwent tumor resection, then PBRT with concurrent TMZ of the Stupp protocol. In total, 10 features, including age, sex, symptom duration, tumor location, WHO grade, surgical intervention, IDH status, MGMT promoter status, CTVhr volume, and CTVlr volume, were collected from each patient. The demographics, molecular markers and PBRT information of the dataset are detailed in **Table 1**.

**Table 1:** Characteristics of all 82 patients, their condition, and treatment.

Characteristics	No. of patients (N = 82, %)
***Sex***	
Male	48 (58.5%)
Female	34 (41.5%)
***Age (years)***	
Median	55.5
Range	19–76
***KPS before radiotherapy***	
>80	64 (78.0%)
≤80	18 (22.0%)
***Tumor Location***	
Lesion involving the SVZ	58 (70.7%)
Lesion not involving the SVZ	24 (29.3%)
***Histology grade (WHO grade)***	
Grade IV	59 (72.0%)
Grade III	23 (28.0%)
***Surgical intervention***	
Partial resection/Biopsy	17 (20.7%)
Subtotal resection	36 (43.9%)
Total resection	29 (35.4%)
***MGMT promoter***	
Methylated	27 (32.9%)
Un-methylated	31 (37.8%)
N/A	24 (29.3%)
***IDH mutation***	
Wild type	16 (19.5%)
Mutant type	66 (80.5%)
***CTVhr volume (cm^3^)***	
Median	98.47
Range	3.72–300.89
***CTVlr volume (cm^3^)***	
Median	220.32
Range	24.00–494.21
***Doses of particle radiation (GyE/fractions)***	
Proton-60GyE/30	48 (58.5%)
Proton-50 GyE/25+ C-ion-10-12GyE/4-5	14 (17.1%)
Proton-60 GyE/30+ C-ion boost to 9–18 GyE/3	18 (22.0%)
Proton-34 GyE/10+ C-ion boost 9 GyE/3*	2 (2.4%)

*For patients ≥ 65 years only. CTVhr, Clinical target volume of high risk; CTVlr, Clinical target volume of low risk; GyE, Gray relative biological equivalent; IDH, Isocitrate dehydrogenase; KPS, Karnofsky Performance Score; MGMT, O[6]-methylguanine-DNA methyltransferase; SVZ, Subventricular zone.

### Survival Analysis of the Entire Cohort of Patients

**Figure 1:**
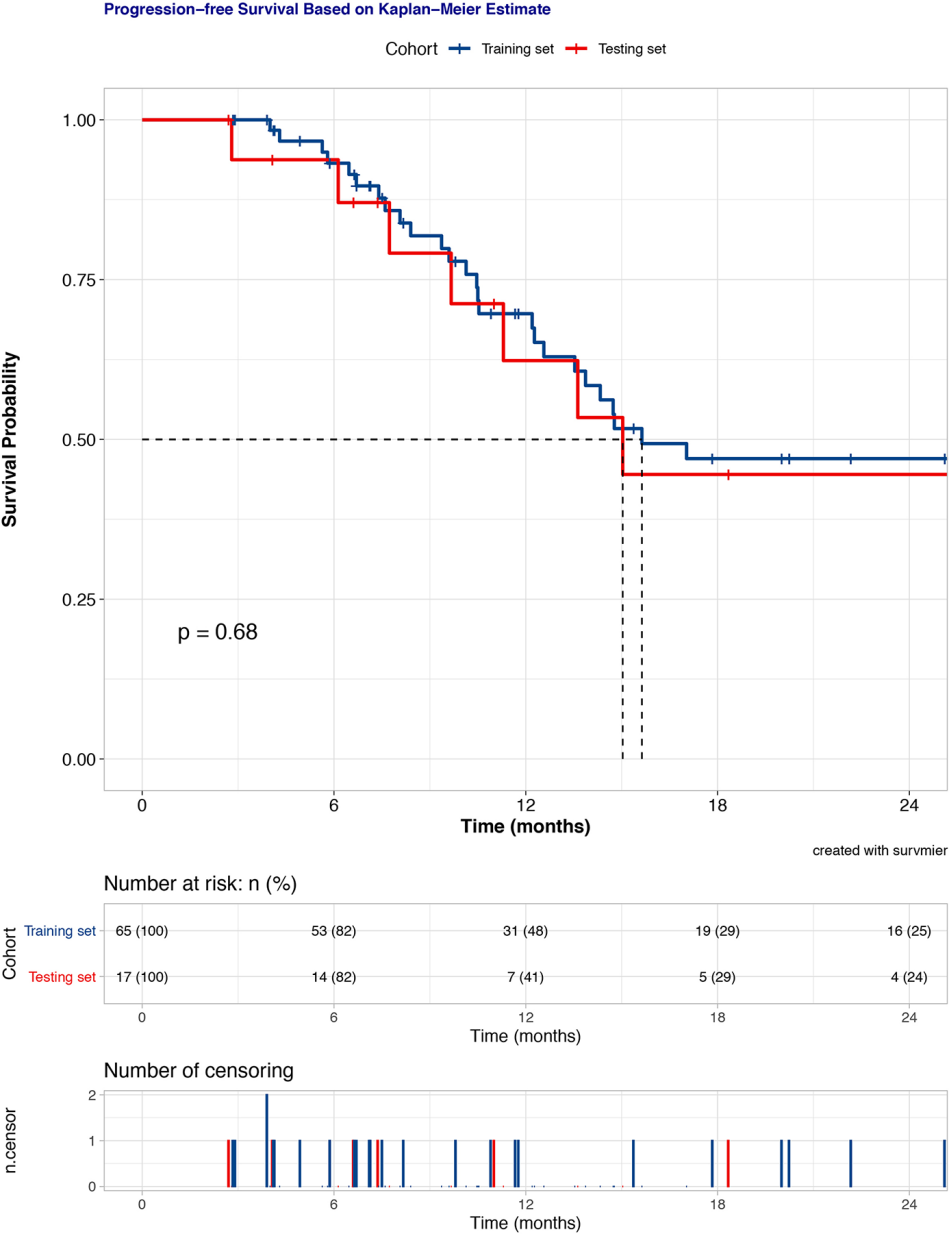
Kaplan-Meier survival curves of progress-free survival for the training and testing set.

The median follow-up period was 16.6 months. At the last follow-up, 37 patients (4 grade III, 33 grade IV) had tumor progression. Progression-free survival (PFS) time was censored for 45 patients (54.9%). The 6-, 12-, and 18-month PFS rates were 93.4%, 68.3%, and 46.6% for the total dataset, respectively. The entire cohort was exclusively split into a training set and a testing set of 65 patients (79.3%) and 17 (20.7%) patients, respectively. No significantly different PFS was revealed between the training and testing datasets by Kaplan-Meier survival curve (P = 0.680, **Figure 1**).

### Comparing the Performance of Cox Proportional Hazard Model With Random Survival Forest

**Figure 2:**
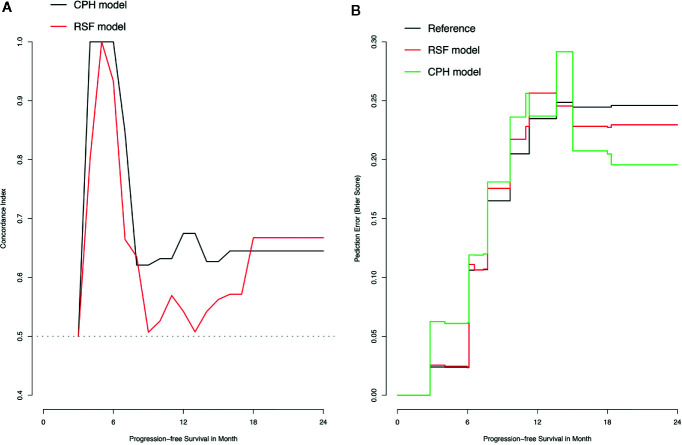
Plots of concordance index (C-index) and brier score (BS) for comparing Cox proportional hazards (CPH) models and random survival forest (RSF) in the testing dataset. **(A)** Plot of C-index; **(B)** Plot of BS.

**Figure 3:**
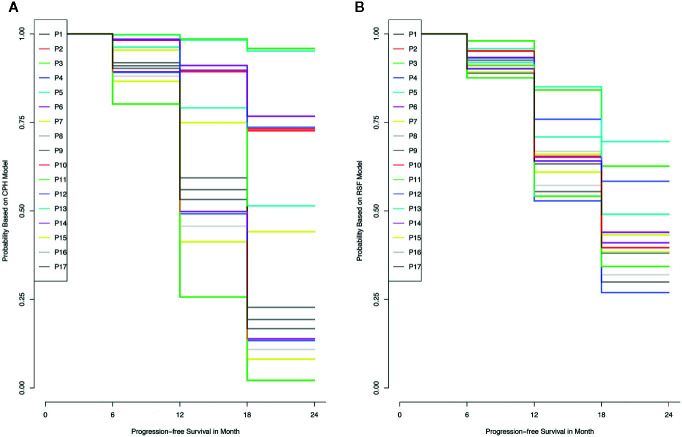
The probability of progress-free survival for each individual in the training data set, according to the results of Cox proportional hazards model **(A)** and random survival forest model **(B)**.

The training set was utilized to build CPH and RSF model. The prediction performance of different models was compared in testing set with both C-index and BS. **Figures 2A, B** respectively illustrated the C-index and BS plots for PFS at various time points. The integrated C-index of CPH and RSF model was 62.9% and 61.1%, respectively. The integrated BS of CPH and RSF was 0.159 and 0.174, respectively (reference = 0.181). **Figure 3** showed the PFS probability with a series time points at 6-, 12-, 18-, and 24- month for each individual in the testing cohort, based on the predicting results of CPH (**Figure 3A**) and RSF (**Figure 3B**) models.

### Identification of Prognostic Factors Using CPH and Random Survival Forest

**Figure 4:**
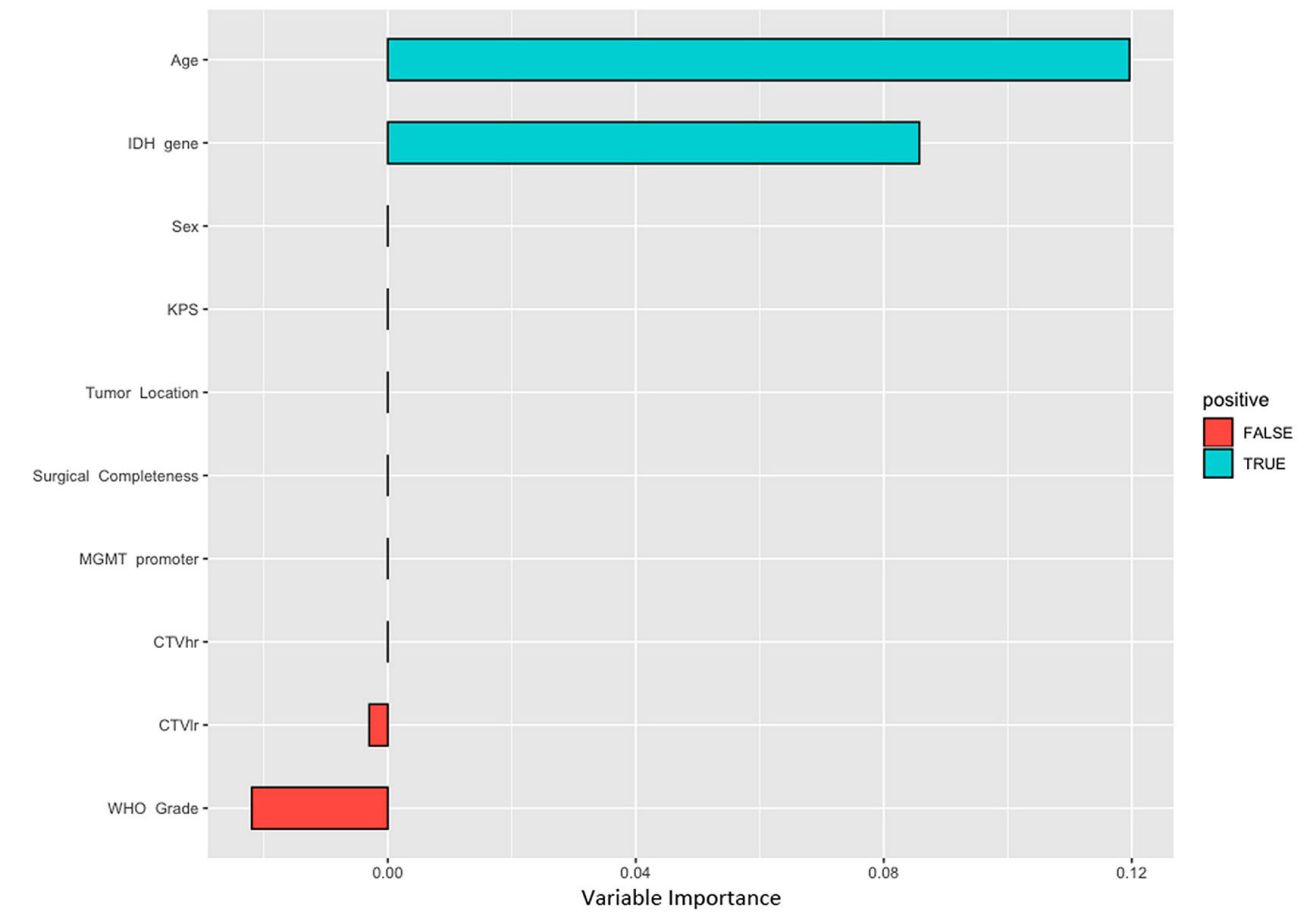
Variable importance of indicated by random forest survival model.

The clinico-pathological features were compared for the correlation to PFS in the training set. According to the CPH model (**Table 2**), univariate analysis documented that age (P = 0.024), WHO grade (P = 0.020), IDH gene (P = 0.019), and MGMT promoter status (P = 0.040) were significantly correlated with PFS; multivariate analysis showed that age (P = 0.041), surgical completeness (P = 0.084), IDH gene (P = 0.057), and MGMT promoter (P = 0.092) had a significant or trend toward the relation with PFS. The RSF model (**Figure 4**) ranked the features in order of importance for PFS, with merely age and IDH status being significantly important variables; meanwhile, tumor grade showed negative importance, meaning that removing a given feature from the model actually improved the performance.

**Table 2:** Cox proportional hazard regressions for progression-free survival in the training set.

Variables^*^	Uni-variate analysis		Mulit-variate analysis	
	HR (95% CI)	P- value	HR (95% CI)	P- value
***Sex***	1.299 (0.616–2.738)	0.492	1.229 (0.510–2.965)	0.646
***Age***	1.040 (1.005–1.075)	0.024	1.040 (1.002–1.081)	0.041
***KPS***	1.665 (0.7356–3.770)	0.221	2.233 (0.788–6.321)	0.131
***WHO Grade***	3.468 (1.200–10.020)	0.020	1.450 (0.418–5.034)	0.559
***Tumor Location***	0.435 (0.162–1.167)	0.098	0.554 (0.178–1.721)	0.307
***Surgical Completeness***	1.177 (0.721–1.922)	0.515	1.835 (0.923–3.647)	0.084
***IDH gene***	4.281 (1.289–14.220)	0.019	4.158 (0.958–18.051)	0.057
***MGMT promoter***	2.387 (1.041–5.472)	0.040	2.555 (0.857–7.622)	0.092
***CTVhr***	1.003 (0.998–1.009)	0.253	1.011 (0.997–1.026)	0.123
***CTVlr***	1.002 (0.999–1.006)	0.236	0.994 (0.985–1.003)	0.195

*The variables were compared in the following ways: sex, female as reference; age as continuous variable; KPS, >80 as reference; WHO grade, grade III as reference; Surgical Completeness, gross total resection as reference; IDH gene, mutant-type as reference; MGMT promoter, methylation as reference; CTVhr (CTVhighrisk), volume as continuous variable; CTVlr (CTVlowrisk), volume as continuous variable. CI, Confidence interval; CTVhr, Clinical target volume of high risk; CTVlr, Clinical target volume of low risk; GyE, Gray relative biological equivalent; HR, Hazard ratio; IDH, Isocitrate dehydrogenase; KPS, Karnofsky Performance Score; MGMT, O[6]-methylguanine-DNA methyltransferase; SVZ, Subventricular zone.

### Nomogram Based on Cox Proportional Hazard Model for Individual PFS Prediction

**Figure 5:**
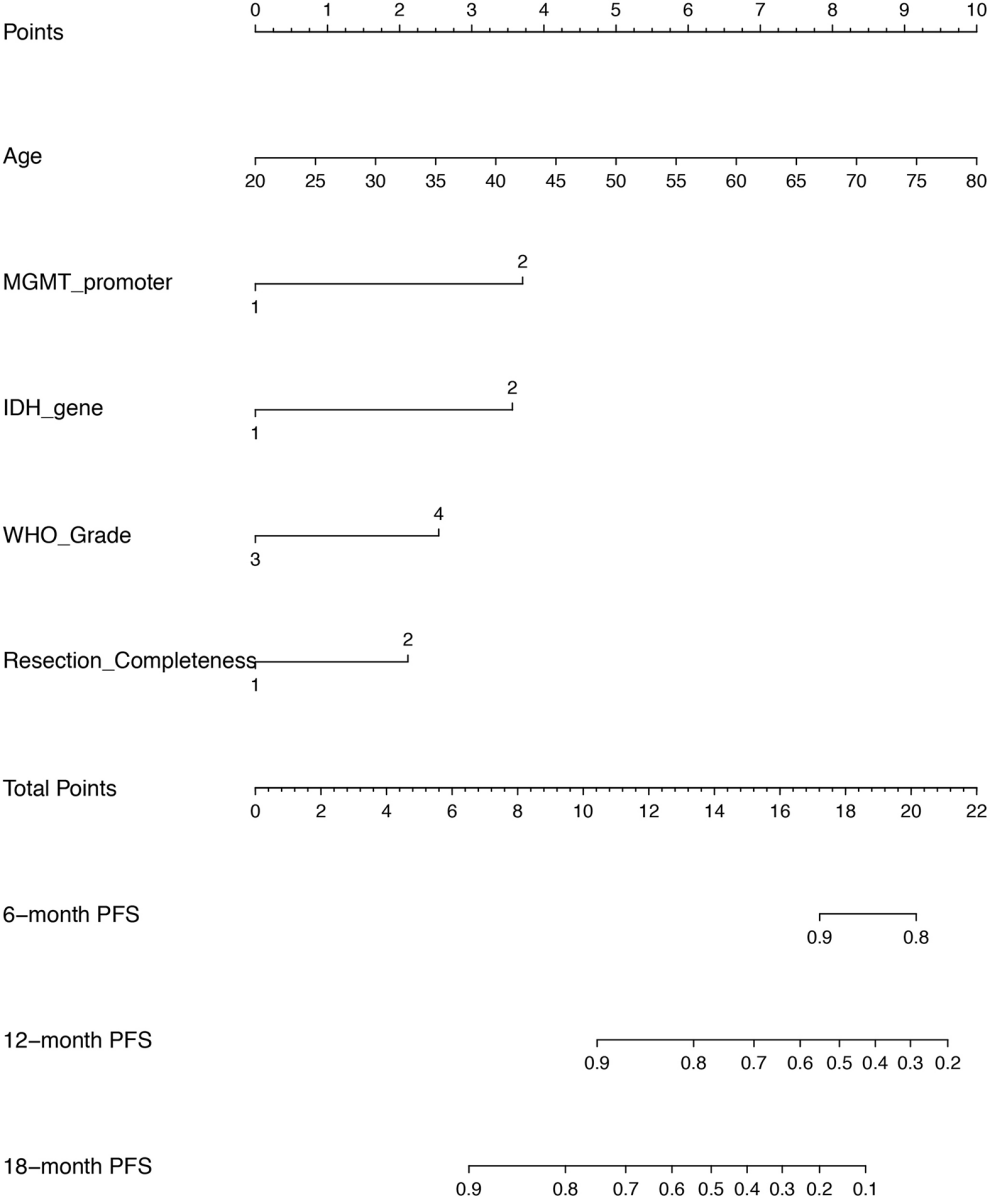
A nomogram of predicting the probability of 6 month-, 12 month-, and 18 month- progression free survival (PFS) at personnel level. The scores of each variable are as follows: age (years) presented as continuous value, MGMT promoter (1 = methylation, 2 = unmethylation/not known), IDH gene (1 = mutant, 2 = wild), WHO grade (3 = Grade III, 4 = Grade IV), resection completeness (1 = gross total resection, 2 = non gross total resection).

Given that the CPH model outperformed RSF model in both discrimination and calibration, a nomogram was built on the base of CPH model to predict the progression probability of HGG patients underwent PBRT at individual level. The variables, including age, MGMT promoter, IDH gene, WHO grade and surgical completeness, that were indicated as significant in univariate analysis or significant (or trend forward) in multivariate analysis, were utilized to conduct the nomogram. In the present nomogram (**Figure 5**), each of the variables was given a point according to hazard ratio (HR). By adding up the total score from each variable and locating it onto the total points scale, the probability of 6-, 12-, and 18- month PFS would be obtained.

## Discussion

Prognosis prediction plays a critical role in clinical and personal decision-making for HGG patients, particularly in the condition of considering the rare source of PBRT as alternative treatment. There have been attempts to conduct traditional statistics and ML methodology to predict individual survival. CPH and RSF model are extensively used in application of cancer survival that generally refers to time-to-event censored data. The main objective of this study was to compare the performance between CPH and RSF models for predicting HGG’s progression underwent PBRT. Our results showed that CPH model present better fit to predict individual PFS in accuracy, precision, and interpretability. Then, we constructed an individual prediction research tool of nomogram based on CPH model for PFS in HGG patients treated with PBRT.

The main advantage of our study is that it approached progression prediction based on a time-to-event dataset. Indeed, there is increasing studies integrating various ML algorithms into improving the predictability of prognosis for cancer. However, most of ML approaches assume that event status is known for all subjects with the utility limited to continuous or binary model. Indeed, right-censored data, referring to the follow-up ends on a subject prior to a patient experiencing an event (i.e., tumor progression or death), is universal in cancer survival. In application of ML approaches analyzing cancer survival, common strategy is to split the patients’ outcome into ordered categorical data based on measuring the disease status at a particular time point. However, this relatively *ad hoc* method does not take the element of time-to-event into account, can merely provide point estimates of outcome and may incur the risk of biasing predication accuracy in the clinical realm (24). The method of RSF utilized in the present study is an extension of random forest for time-to-event data, represents an attractive ML approach that allows for the computation of personnel-level survival prediction through more granular insight and mitigates the systemic bias associated with incomplete follow-up.

Another advantage of our study is that the performances of different models were graphically compared with a comprehensive aspect of discrimination and calibration at various time points, rather than a fixed time. Discrimination represents the ability of a model to separate observations on subjective-level, whereas calibration is a descriptor of a predictive model that characterizes the agreement between the observed and predicted outcome on a population level. C-index, the main metric quantified of discrimination in this study, reflects the probability that for a random selection of any 2 HGG patients, the patient with earlier tumor progression is ranked with higher risk of progression according to the model. Hence, the C-index takes into account of both the occurrence of the event and the length of follow-up and is particularly well suited for time-to event data analysis. Indeed, any model (i.e., CPH model) with the ability to forecast properly ordered but proportional event times can score high value of C-index (25). Hence, the evaluation of calibration, another metric of prediction accuracy, is essential but unfortunately under-explored in time-to-event models for many studies. Even in studies that performed the assessment of calibration, the method of a calibration plot can only provide information at a specific time point (e.g., 1-year survival probability). Here, in our study, we presented a measurement of BS plot to assess the model performance at various time points. In precise, the BS measures the mean squared difference between the predicted progression probability and the actual outcome for all HGG patients at group level. Note that BS takes on a value between 0 and 1, and the lower of BS indicates that better predictions are calibrated.

The most important finding of the present study is that CPH outperformed RSF with both C-index and BS in the predictability of progression for HGG patients underwent PBRT in a relatively small sample. In terms of C-index, our result was consistent with a study enrolling 289 cases as a testing set and 98 cases as a validation test conducted by Gittleman et al. (16), in which C-index for predicting survival of lower-grade glioma at 60, 90, and 120 months were measured for CPH (0.844, 0.843, 0.841) and RSF (0.806, 0.791, 0.782), respectively. However, it is still controversial which method can consistently achieve better accuracy of predicting prognosis *via* the measurement of C-index in glioma. Audureau et al. (26) conducted a retrospective multi-centric study enrolling 777 patients with recurrent glioblastoma, split into a training set of 407 cases and an external validation set of 370 cases; the results presented the discrimination C-indexes of CPH and RSF as 69.80% and 70.14% in the external validation set, respectively. Based on a larger population from the Surveillance, Epidemiology, and End Results (SEER) database that comprised 20,821 glioblastoma cases split into a training and validation test set with an 80/20 ratio, Senders et al. (14) revealed the integrated C-index of CPH and RSF as 0.69 and 0.68, respectively. On the hands of model calibration, neither of the studies performed such analysis. All these three studies lack the assessment of BS, or any other methods of calibration, for the comparison of CPH and RSF. Our study, to our knowledge, is the first study to directly compare the discrimination and calibration for CPH and RSF in glioma.

In looking at variables with significant relation to survival in our results, CPH model documented that age, MGMT promoter, surgical completeness, IDH gene and KPS had a significant or trend toward relation with tumor progression. In comparison, only IDH status and age were indicated with significant importance affecting PFS according to the RSF model. It should be noted that all the significant variables in our CPH model are well-known prognostic factors in the clinical decision-making of HGG. In brief, the CPH model identified more statistically significant prognostic factors that are generally considered important in decision making in a clinical setting. Hence, we believe that CPH model had a better interpretability as compared to RSF in terms of exploring the critical factors for predicting tumor progression after PBRT. There is a possibility that the relatively small sample and/or the different mathematical underpinnings contributed to this different effect. In principle, RSF model is based on searching for the best variables used to split the node by maximizing the log-rank methods, and the variable importance refers to a measurement of the increase of predicting error when perturbation is added to the variable. While in CPH model, the importance of variables can be interpreted as HR and P-value. Indeed, one common drawback of RSF is a bias toward inclusion of variables with many split points that may lead to a bias in resulting summary of variable importance (27–29).

We also constructed a nomogram based on CPH model due to its superior performance over RSF model. As a pictorial representation that uses various potential prognostic markers to depict a scoring model, nomogram is provided as a visual tool to generate a probability of a clinical outcome for a given individual. Patients’ survival-related nomograms for HGG have been developed in series of studies (30–35), but with a common drawback that some critical patient-related, tumor-related or treatment-related information were not incorporated. Moreover, all these studies were only applied for patients treated with photon-based radiotherapy, but not PBRT. In the context of PBRT, previous results from Germany and Japan showed that photon radiation combined with carbon ion boost improved the outcome of HGG patients (3); and recently, we reported our early experience with an encouraging efficacy of PBRT in HGG (4). It is well known that PBRT has been increasingly spread worldwide to treat cancer, and great expectation has been placed with PBRT to improve the dismal outcome of HGG. The present nomogram in our study can provide a tool of reference for counseling PBRT as a treatment option for HGG based on common-used prognostic markers, and may be informative of future precise medicine of PBRT in HGG.

There are several limitations of this study to be discussed. First, due to the retrospective nature of this study, our results were derived from a relatively limited observation database that may introduce some inevitable bias. Statistically, it is of better generalizability to compare methods with a prospective design, or at least external validation dataset. Meanwhile, with a phase III clinical trial going in our institution (36), the present study provided a blueprint of methodology to perform a prospective validation in the future. Second, our study was designed as a purely academic research to compare the prediction performance of CPH and RSF in discrimination, calibration and interpretability. The present nomogram based on CPH model should not be directly implemented in the clinical practice prior to a prospective validation. Nevertheless, based on our results, we recommend evaluating fitted ML models on several criteria rather than a singular focus on prediction accuracy. Third, though the variables in our studies consisted of systematical information occupying important role in the management of HGG, it should be noted that the critical issue of inherent high heterogeneity within HGG could not be well settled through these features. Noticeably, radiomic, particularly referring to functional brain imaging technique, can provide an integrative and dynamic view of the whole tumor tissue and serve as a reliable tool to tackle the issue of heterogeneity. In this context, our ongoing phase III trial adopts multi-modal imaging, including MET-PET, perfusion weighted imaging (PWI), diffusion tensor imaging (DTI), and MR spectroscopy (MRS), for each patient (36). Thus, more comprehensive models that include imaging parameters will be assessed in the future.

## Conclusion

This study indicated a superior accuracy of CPH as compared to RSF in a relatively small sample data of HGG patients for predicting tumor progression after PBRT. As more approaches about ML techniques are implemented to glioma prognostication purposes, comprehensive criteria with discrimination and calibration, as well as interpretability, is recommend in evaluating fitted models for clinical deployment.

## Data Availability Statement

The raw data supporting the conclusions of this article will be made available by the authors, without undue reservation.

## Ethics Statement

The studies involving human participants were reviewed and approved by Shanghai Proton and Heavy Ion Center. The patients/participants provided their written informed consent to participate in this study.

## Author Contributions

Conception and design: LK and JL. Acquisition of data: XQ, JG, JY, JH, and WH. Analysis and interpretation of data: XQ, JG, and JH. Drafting or revising the article: XQ, LK, and JL. All authors contributed to the article and approved the submitted version.

## Funding

The National Key Research and Development Program of China (Project No. 2018YFC0115700 and 2017YFC0108603); The Shanghai Academic/Technology Research Leader Program (Project No. 19XD1432900 and 18XD1423000); Shanghai Hospital Development Center (Joint Breakthrough Project for New Frontier Technologies. Project No. SHDC12016120); Science and Technology Commission of Shanghai Municipality (Project No. 19411951000); Science and Technology Development Fund of Shanghai Pudong New Area (Project No. PKJ2018-Y51, PKJ2017-Y50 and No.PKJ2017-Y49).

## Conflict of Interest

The authors declare that the research was conducted in the absence of any commercial or financial relationships that could be construed as a potential conflict of interest.
